# Psychometric evaluation of the Indolent Systemic Mastocytosis Symptom Assessment Form (ISM-SAF^©^) and determination of a threshold score for moderate symptoms

**DOI:** 10.1186/s13023-023-02661-1

**Published:** 2023-03-25

**Authors:** Alan L. Shields, Fiona Taylor, Roger E. Lamoureux, Brad Padilla, Kas Severson, Tanya Green, Anthony L. Boral, Cem Akin, Frank Siebenhaar, Brenton Mar

**Affiliations:** 1 Adelphi Values, Boston, MA USA; 2 Blueprint Medicines, Cambridge, MA USA; 3 University of Michigan, Ann Arbor, MI USA; 4 Institute of Allergology, Charité - Universitätsmedizin Berlin, corporate member of Freie Universität Berlin, Humboldt-Universität Zu Berlin, and Berlin Institute of Health, Berlin, Germany; 5 Fraunhofer Institute of Translational Medicine and Pharmacology ITMP, Immunology and Allergology IA, Berlin, Germany

**Keywords:** Psychometric evaluation, Instrument development, Patient-reported outcomes, Indolent systemic mastocytosis

## Abstract

**Background:**

The Indolent Systemic Mastocytosis Symptom Assessment Form (ISM-SAF) (©Blueprint Medicines Corporation), a 12-item daily diary that assesses 11 signs and symptoms of indolent systemic mastocytosis (ISM) and smoldering systemic mastocytosis (SSM), was psychometrically evaluated among patients with ISM. Additionally, thresholds of the ISM-SAF total symptom score (TSS) to distinguish patients with moderate to severe symptoms from those with mild symptoms were evaluated.

**Methods:**

The ISM-SAF was completed daily as an electronic diary in a prospective, observational study utilizing an online survey of patients with ISM in the United States. Descriptive statistics, psychometric analyses, and analyses to estimate ISM-SAF TSS clinical cutoff values were conducted.

**Results:**

A total of 103 patients (81.6% female; mean age = 50.2 [± 12.6]) with a self-reported diagnosis of ISM or SSM (58 of whom also had a medically documented diagnosis) contributed to the analyses. Psychometric analysis supported the trustworthiness of the biweekly TSS, which was reliable (*α* > 0.8, ICC > 0.9), construct-valid, and able to distinguish among clinically distinct groups as specified by the Patient Global Impression of Severity, 12-item Short-Form Health Survey, and Mastocytosis Quality of Life Questionnaire (*p* < 0.01). A biweekly ISM-SAF TSS from 21 to 28 begins to distinguish the moderately to severely symptomatic ISM/SSM patients from mildly symptomatic patients.

**Conclusion:**

The biweekly TSS of ISM-SAF was reliable, construct-valid, and able to distinguish among clinically distinct groups. A cut-off value of 28 is a conservative threshold that can be used for screening purposes in future clinical studies to identify patients with at least a moderate severity of ISM symptoms.

**Supplementary Information:**

The online version contains supplementary material available at 10.1186/s13023-023-02661-1.

## Introduction

Systemic mastocytosis is a rare, clonal mast cell neoplasm driven by the KIT D816V mutation [1] that is characterized by uncontrolled proliferation and activation of mast cells, which leads to severe and unpredictable symptoms for patients with systemic mastocytosis [2]. As a rare disease, the incidence of all systemic mastocytosis subtypes is approximately 0.89 per 100,000 per year [3] and the prevalence of indolent systemic mastocytosis (ISM) in the Groningen region of the Netherlands, a major referral area for systemic mastocytosis patients, is estimated at 13/100,000 [4]. Unlike other forms of systemic mastocytosis, ISM is associated with a normal or near-normal life-expectancy [5]; however, many ISM patients experience severe, life-limiting symptoms that significantly impact daily life [6, 7]. Smoldering systemic mastocytosis (SSM) is similar to ISM in its symptomatology, but is associated with a relatively higher burden of mast cells, and was considered a rare subtype of ISM prior to the 2016 WHO reclassification of systemic mastocytosis [8]. Unfortunately, there are limited treatment options available for patients with systemic mastocytosis and no approved therapies for patients with ISM [8].

As drug sponsors develop ISM treatments, the availability of well-defined and reliable patient-reported outcome (PRO) questionnaires to assess clinical benefit as a result of those interventions are important. However, no such instrument yet exists or was considered to be consistent with Food and Drug Administration (FDA) regulatory guidelines for use in the ISM patient population [9–12]. To fill this gap, the Indolent Systemic Mastocytosis Symptom Assessment Form (ISM-SAF)(©Blueprint Medicines Corporation) was developed in ways consistent with regulatory [9] and scientific guidelines [10, 13] to evaluate clinical benefit hypotheses for use in product approval and labeling decisions.

The content validity of ISM-SAF was established [14], as evidenced by a variety of qualitative research inquiries along with feedback from the FDA to ensure the ISM-SAF aligned with regulatory expectations for instruments intended for use in clinical trials [9]. The goal of the present study was to perform an exploratory psychometric evaluation of scores produced by the ISM-SAF and to explore its use as a clinical trial screening tool. The psychometric performance of scores produced by the ISM-SAF among patients who have ISM or SSM with respect to score variability, distribution, missingness, reliability, and construct-related validity was evaluated to provide evidence for the trustworthiness of the ISM-SAF scores. Additionally, this study aimed to establish an ISM-SAF total symptom score (TSS) cutoff value (i.e., a severity cutoff point) that could distinguish patients with moderate to severe symptoms relative to those with less severe symptoms; subsequently, the ISM-SAF could be used to screen patient eligibility for clinical studies assessing symptomatic improvement based on a minimum level of sign and symptom severity.

## Methods

### Study design

A prospective, non-interventional, observational study utilized an online survey of patients in the United States diagnosed with ISM or SSM, who completed PRO assessments using a web-based electronic platform (SurveyMonkey^®^) over the course of 15 days. All study documents were submitted to and approved by a centralized institutional review board (IRB), Schulman IRB, prior to initiating patient recruitment.

Patients were identified through advertising by The Mastocytosis Society, a patient advocacy group for individuals with mastocytosis and other mast cell disorders. The target sample size for this study was 75 adult patients (age ≥ 18) with ISM or SSM. When interested individuals clicked on the web-enabled link in the study advertisement or study recruitment email, they were directed to a web-based, Health Insurance Portability and Accountability Act-compliant [15] platform (SurveyMonkey^®^) to provide electronic informed consent using an informed consent form [16, 17]. Patient eligibility was confirmed via a patient screener. Participants with a self-reported diagnosis of ISM or SSM were recruited for study participation. Individuals were excluded from the study if they self-reported mast cell activation syndrome, advanced systemic mastocytosis, or any other hematologic malignancies/blood cancers. Additionally, all participants were asked to provide medical documentation of their ISM or SSM diagnosis. Participants unable to provide medical documentation of diagnosis were still allowed to participate; however, a separate analysis was performed for participants whose ISM or SSM diagnosis was confirmed based on a physician review of medical records. Patients were then provided with Day 1 assessments within 48 h. Specifically, patients were asked on Day 1 to provide demographic and health information and complete the following PRO assessments: ISM-SAF, Patient Global Impression of Severity (PGIS), 12-Item Short Form Survey, Version 2 (SF12v2^®^), and Mastocytosis Quality of Life Questionnaire (MC-QoL). Subsequently, patients were asked to complete the ISM-SAF on each of the ensuing 13 days (Day 2–Day 14), followed by completion of the ISM-SAF, PGIS, SF12v2^®^, and MC-QoL on Day 15.

### Analysis populations

The analysis populations included two cohorts. The first cohort included all patients who self-reported a diagnosis of ISM or SSM (Self-reported Diagnosis Cohort), and the second cohort included the subsample of patients who also provided a confirmed diagnosis of ISM or SSM via medical documentation (Medically Documented Diagnosis Cohort). Test–retest reliability for the ISM-SAF scores was evaluated using a subsample of patients who exhibited no change in PGIS from Day 1 to Day 15. Post-hoc reliability and validity analyses were performed on patients with only a self-reported diagnosis (i.e., without medical documentation) to give confidence that the scores were similar between patient samples.

### Study assessments

#### ISM-SAF

The ISM-SAF is a 12-item daily diary that assesses the severity of 11 ISM symptoms including bone pain, abdominal pain, headache, nausea, spots, itching, flushing, fatigue, dizziness, brain fog, and diarrhea over a 24-h recall period with an 11-point numeric rating scale (NRS), where 0 = No [symptom] and 10 = Worst imaginable [symptom]; the twelfth item assesses diarrhea frequency by asking patients to enter a discrete numerical value. As a once-daily diary, the ISM-SAF was completed daily from Day 1 to Day 15 on the study’s web-based platform.

The ISM-SAF is scored at an item level, domain level, and total score level. Two severity domains were hypothesized: the Gastrointestinal Symptom Score (GSS), composed of abdominal pain, nausea, and diarrhea severity (score range 0–30), and the Skin Symptom Score (SSS), composed of spots, itching, and flushing severity (score range 0–30). The daily domain scores are generated by summing the item scores of each day, and all contributing items need to be completed to calculate a daily score. The daily total symptom score (TSS) was created by combining all items except the diarrhea frequency item (range 0–110). Weekly scores were derived as seven-day averages of daily scores (Week 1: Days 2–8, Week 2: Days 9–15, with a minimum of four daily scores required), and biweekly scores were derived by averaging scores over 14 days (Days 2–15, with a minimum of seven daily scores required).

### Supportive measures

The psychometric evaluation of the ISM-SAF was supported by other clinical and PRO assessments, which were administered on Day 1 and Day 15:

#### Patient Global Impression of Severity (PGIS)

The PGIS is a single item that asks patients to rate their overall symptom severity at present on a five-point scale (“0– absent,” “1–minimal,” “2–moderate,” “3–severe,” and “4–very severe”).

#### SF-12v2^®^ Health Survey (SF-12v2^®^)

The SF-12v2^®^ is a 12-item PRO questionnaire assessing physical and emotional health- and function-related limitations using a recall period of “the past week” on three- and five-point verbal response scales (scores range from 0 to 100, with higher scores representing better health) [18, 19]. It comprises eight health domains (physical functioning, role physical, bodily pain, general health, vitality, social functioning, role emotional, and mental health) and composite scores are calculated for mental and physical constructs.

#### Mastocytosis Quality of Life Questionnaire (MC-QoL)

The MC-QoL is a 27-item PRO questionnaire assessing health-related quality of life impairment in patients with cutaneous mastocytosis and ISM [20] using a recall period of “the past two weeks” and a five-point verbal response scale (scores ranges from 0 to 100, where higher scores indicate higher health-related quality of life impairment). It consists of four domains (symptoms, emotions, social life/functioning, and skin) and a total score is calculated.

### Analyses

#### Sample

Descriptive statistics for age, gender, race, ethnicity, work status, and education level; experience of mastocytosis in the skin; and treatment history were computed and presented for the study sample upon entry into the study.

#### Score distribution

Item-level and domain-level score distributions for the ISM-SAF were evaluated in terms of respondents’ use of the entire scale and for floor and ceiling effects.

#### Reliability

Reliability estimates characterize consistency and reproducibility of scores produced by a questionnaire when administered to a particular target patient population and in a particular context of use and can be evaluated using various methods, depending on the nature of the assessment and context of administration. In this study, the reliability of ISM-SAF scores was assessed in two ways. First, internal consistency reliability was investigated by calculations of Cronbach’s alpha coefficient (α, range 0 to 1) for the TSS, GSS, and SSS (biweekly scores) and again with each item removed to assess the impact that removal had on the overall α. Scores greater than 0.70 are typically seen as sufficient for research purposes [21]. Second, test–retest reliability was assessed among patients who exhibited no change in PGIS from Day 1 to Day 15, using the intra-class correlation coefficient (ICC) [22] and its 95% confidence internal, and based on the comparison of ISM-SAF TSS, domain, and item scores collected during Week 1 and Week 2.

#### Construct-related validity

Construct-related validity is concluded upon evidence that scores produced by a target questionnaire relate to scores from other assessments in ways that are logical and according to a priori hypotheses [9]. In the present study, the relationships between ISM-SAF scores and those generated by the supportive assessments were examined via correlational analysis and interpreted based on the following absolute value guidelines (correlation range is -1 to 1): negligible relationship, r = 0.0–0.09; small relationship, r = 0.1–0.29; medium relationship, r = 0.30–0.49; and strong relationship, r ≥ 0.50. [23, 24]

Known-groups methods characterize the degree to which a PRO questionnaire generates scores capable of distinguishing among patient groups hypothesized to be clinically distinct [9]. This analysis was conducted using the PGIS, MC-QoL (tertiles), and SF-12v2^®^ (tertiles) to categorize patients into “known groups” on Day 15, and ISM-SAF scores were described across patient severity groups. It was hypothesized that the higher ISM-SAF scores (greater symptoms) would be associated with groups of patients with higher PGIS and MC-QoL scores and lower SF-12v2^®^ scores.

Daily, weekly, or biweekly TSS and domain scores were used in correlational and known-groups analyses to match the recall period of the respective supportive assessment administered on Day 15 (i.e., PGIS correlation with Day 15 ISM-SAF scores, SF-12v2^®^ correlation with Week 2 ISM-SAF scores, and MC-QoL correlation with biweekly ISM-SAF scores).

#### ISM-SAF score severity cutoffs

To estimate a cutoff value in the ISM-SAF TSS to identify respondents who experience moderate to severe signs and symptoms of ISM, tertile groupings were formed and receiver operating characteristic (ROC) analyses were conducted. Tertile groupings of the biweekly TSS were calculated for both the Self-reported Diagnosis Cohort and the Medically Documented Diagnosis Cohort. ROC curve analysis was conducted to separate patients who were minimally symptomatic from patients who were moderately or more severely symptomatic based on the dichotomized biweekly PGIS scores at Day 15 (i.e., patients with a score of one or below on the PGIS were defined as having minimal or absent symptom severity [coded as 0], and patients with a score of two or above were identified as having some level of symptom severity [coded as 1]). Individual TSSs were examined with regard to sensitivity (i.e., the degree to which the score would correctly identify individuals with moderate to severe symptoms) and specificity (i.e., the degree to which the score would correctly identify individuals who did not have moderate to severe symptoms). Positive and negative predictive values (PPV and NPV) indicated the degree to which the score identified individuals who were also classified as moderate or severe/very severe versus absent/minimal on the PGIS, respectively. The cutoff point on the TSS with the largest Youden’s index indicated the maximization of sensitivity and specificity.

## Results

### Study sample

A total of 116 eligible patients were screened into the study; 103 were included in the Self-reported Diagnosis Cohort, and 58 were included in the Medically Documented Diagnosis Cohort (ISM: n = 56, 96.6%; SSM: n = 2, 3.4%). In the Self-reported Diagnosis Cohort, mean age was 50.2 years (standard deviation [SD] = 12.6), 81.6% were female, and 98.1% were white. Demographic characteristics for the Medically Documented Diagnosis Cohort were largely similar, with a slightly lower proportion of male patients compared to the Self-reported Diagnosis Cohort (10.3% versus 18.4%). Complete demographic and health information details for both cohorts are presented in Table 1; Additional file 1: Table S1 additionally contains demographic and health information for those patients with only a self-reported diagnosis (n = 45). Concomitant medications reported by patients on entry into the study are presented in Table 2.

**Table 1:** Sample demographic and health characteristics

Demographic or health characteristic	Self-reported diagnosis cohort (N = 103) Statistic or n (%)	Medically documented diagnosis cohort (n = 58) Statistic or n (%)
*Age at day 1 (in years)*		
Mean (SD)	50.2 (12.6)	48.9 (13.3)
Median	49.3	47.4
Min–max	18.6–76.1	18.6–72.2
Missing/no response	7	4
*Gender*		
Female	84 (81.6%)	52 (89.7%)
Male	19 (18.4%)	6 (10.3%)
*Race*		
White	101 (98.1%)	58 (100.0%)
Other^a^	2 (1.9%)	0 (0.0%)
*Ethnicity*		
Hispanic or latino	6 (5.8%)	1 (1.7%)
Not Hispanic or latino	97 (94.2%)	57 (98.3%)
*Work status*		
Working full-time	45 (43.7%)	21 (36.2%)
On disability^b^	19 (18.4%)	11 (19.0%)
Working part-time^c^	18 (17.5%)	14 (24.1%)
Retired	14 (13.6%)	7 (12.1%)
Other^d^	4 (3.9%)	3 (5.2%)
Unemployed	2 (1.9%)	1 (1.7%)
Student	1 (1.0%)	1 (1.7%)
*Highest level of education*		
High school diploma (or GED) or less	3 (2.9%)	2 (3.4%)
Some college or certificate program	29 (28.2%)	15 (25.9%)
College or university degree (two- or four-year)	44 (42.7%)	24 (41.4%)
Graduate degree	27 (26.2%)	17 (29.3%)

^a^Other race includes patients who characterized themselves as “Hispanic” and “Puerto Rican”

^b^On disability includes those that are awaiting a disability hearing/decision

^c^Part-time is characterized as work that is not consistently done five days a week

^d^Other work statuses include patients who noted that they are self-employed or work from home but did not indicate how much time per week they are working

**Table 2:** Concomitant medication use (Self-reported Diagnosis Cohort; N = 103)

Treatment	Currently taking^*^
*H1 antihistamines*	
Loratadine/Claritin	14 (13.6%)
Diphenhydramine/Benadryl	47 (45.6%)
Cetirizine/Zyrtec	50 (48.5%)
Fexofenadine/Allegra	35 (34.0%)
Hydroxyzine/Vistaril/Atarax	23 (22.3%)
*H2 antihistamines*	
Cimetidine/Tagamet	1 (1.0%)
Famotidine/Pepcid	22 (21.4%)
Ranitidine/Zantac^†^	61 (59.2%)
*Proton pump inhibitors*	
Omeprazole/Prilosec	18 (17.5%)
Pantoprazole/Protonix	8 (7.8%)
*Leukotriene inhibitors*	
Montelukast/Singulair	39 (37.9%)
Zafirlukast/Accolate	3 (2.9%)
*Oral glucocorticoids*	
Prednisone/Deltasone	5 (4.9%)
*Cromolyn sodium*	
Cromoglicic acid/Nasalcrom/Gastrocrom	36 (35.0%)
*Anti-IgE*	
Omalizumab/Xolair	12 (11.7%)
*Cytoreductive agents*	
Hydroxyurea/Hydrea	1 (1.0%)
Interferon alpha/IFN/Roferon A/Intron A/Multiferon	1 (1.0%)
Imatinib/Gleevec	2 (1.9%)
Midostaurin/PKC412/Rydapt	1 (1.0%)
*Psoralen plus UV phototherapy*	
PUVA	1 (1.0%)
*Bisphosphonates for osteoporosis*	
Alendronate/Aledronic acid/Fosamax	5 (4.9%)
Risedronate/Risedronic acid/Actenol/Atelvia	2 (1.9%)
Pamidronic acid/Aredia	2 (1.9%)
Zoledronic acid/Reclast/Zometa	3 (2.9%)
*Epinephrine for allergic reactions*	
Adrenalin/EpiPen	12 (11.7%)

*Patients may be taking more than one medication

^†^Ranitidine/Zantac is no longer available due to contamination issues

### Score distribution

Descriptive analysis of the ISM-SAF indicated that while patients used the range of response options available to them for each item (i.e., 0 to 10), not all patients reported experiencing all symptoms and, when symptoms were reported, severity rates were variable. In the Self-reported Diagnosis Cohort, the mean weekly GSS, SSS, and TSS were 5.3 (SD = 4.5), 8.3 (SD = 5.3), and 27.3 (SD = 15.4), respectively. The mean of weekly ISM-SAF items ranged from 1.4 (SD = 1.8) to 4.6 (SD = 2.4), which were all lower than 50% of the scale. It is notable that responses tended to cluster near the lower end of the scale (i.e., less severe symptom experience) and many patients reported “no [symptom]” (i.e., a response choice of “0”). The same pattern was observed in the Medically Documented Diagnosis Cohort.

### Reliability

#### Internal consistency reliability

Internal consistency estimates (α) are presented in Table 3 and suggest adequate reliability for use in research settings for the TSS and marginal to adequate reliability for the GSS and SSS as a biweekly score in both cohorts. Removal of items from the TSS typically reduced overall alpha coefficients; any instances in which alpha increased (e.g., Item 4, spots) were only marginal. Additional file 1: Table S2 presents internal consistency reliability estimates for those patients with only a self-reported diagnosis.

**Table 3:** Internal consistency reliability (α) on the biweekly ISM-SAF ^©^ total symptom scale and domain scores and test–retest reliability between Weeks 1 and 2 on Patient Global Impression of Severity stable patients (n = 61)

	Biweekly score (Days 2–15)		Between weeks 1 and 2
	Self-reported diagnosis cohort (n = 103)	Medically documented diagnosis cohort (n = 58)	Test–retest analysis population^b^
	Coefficient alpha^a^	Coefficient alpha^a^	Reliability estimate^c^ (95% confidence interval)
*Domain/total score*^*d*^			
TSS	0.884	0.876	0.962 (0.936–0.977)
GSS	0.777	0.685	0.936 (0.894–0.962)
SSS	0.667	0.700	0.962 (0.937–0.977)
*Items*	*Alpha of TSS if item removed*		
Item 1: bone pain	0.870	0.862	0.943 (0.905–0.966)
Item 2: abdominal pain	0.866	0.859	0.922 (0.870–0.953)
Item 3: nausea	0.870	0.861	0.937 (0.895–0.962)
Item 4: spots	0.896	0.881	0.974 (0.957–0.985)
Item 5: itching	0.875	0.866	0.902 (0.837–0.941)
Item 6: flushing	0.870	0.859	0.971 (0.952–0.983)
Item 7: fatigue	0.861	0.849	0.951 (0.918–0.971)
Item 8: dizziness	0.868	0.859	0.929 (0.881–0.957)
Item 9: brain fog	0.876	0.867	0.956 (0.926–0.973)
Item 10: headache	0.871	0.861	0.905 (0.841–0.943)
Item 11: diarrhea (frequency)	–	–	0.885 (0.809–0.931)
Item 12: diarrhea severity	0.883	0.887	0.869 (0.781–0.921)

^a^Only coefficient alpha for the ISM-SAF^©^ domain scores presented (i.e., item to domain score correlation and coefficient alpha with item removed are not presented here). The Cronbach’s alpha presented for each item is the α of the TSS if the item was removed

^b^Test-retest reliability for the ISM-SAF^©^ scores was evaluated using a test–retest analysis population including patients who exhibited no change in PGIS from Day 1 to Day 15

^c^The reliability estimates provided for the TSS and domain scores are ICCs computed using Shrout-Fleiss reliability ICC (3,k): two-way mixed multiple measure

^d^The ISM-SAF^©^ item score ranges from 0 to 10, while the domain and total scores (GSS, SSS, and TSS) range from 0 to 30, 0 to 30, and 0 to 110, respectively; for all score types, higher scores are associated with a higher level of symptom severity

#### Test–retest reliability

Test–retest reliability estimates comparing Week 1 (an average of scores generated on Days 2 to 8) and Week 2 (an average of scores generated on Days 9 to 15) were all excellent (> 0.75) [25] based on patients who exhibited no change in PGIS scores from Day 1 to Day 15 (n = 61) (Table 3).

### Validity

#### Construct-related validity

The relationships between the TSS and other variables were strong and in the expected direction. No noteworthy differences or distinctions were observed regarding the pattern of relationships among the Self-reported Diagnosis Cohort and the Medically Documented Diagnosis Cohort. As expected, the TSS was more strongly correlated with variables assessing symptoms and physical function (such as the role physical and bodily pain domains of the SF-12v2^®^ and the symptoms domain of the MC-QoL) and less strongly correlated with variables associated with more distal disease impacts (such as the mental component score or the role emotional domain of the SF-12v2^®^). Patients reporting increased symptom involvement on the ISM-SAF also rated themselves as more severely afflicted on the PGIS. Correlations with other measures were generally greater for the TSS than for the GSS and SSS, except for the MC-QoL Skin domain, which correlated most strongly with the SSS as expected (Table 4). Additional file 1: Table S3 presents comparable data for those patients with only a self-reported diagnosis.

**Table 4:** Spearman correlations of ISM-SAF total and domain scores with other measures administered at Day 15

Concurrent measure	Self-reported diagnosis cohort (N = 103)			Medically documented diagnosis cohort (n = 58)		
	TSS	GSS	SSS	TSS	GSS	SSS
SF-12: physical functioning	− 0.585	− 0.480	− *0.265*	− **0.685**	− 0.530	− 0.484
SF-12: role physical	− **0.741**	− **0.608**	− 0.390	− **0.729**	− 0.547	− 0.528
SF-12: bodily pain	− **0.722**	− 0.557	− 0.418	− **0.760**	− 0.514	− 0.585
SF-12: general health	− 0.560	− 0.417	− 0.329	− **0.667**	− 0.432	− 0.511
SF-12: vitality	− 0.504	− 0.441	− *0.212*	− 0.453	− 0.305	− *0.222*
SF-12: social functioning	− 0.584	− 0.568	− 0.317	− 0.577	− 0.505	− 0.408
SF-12: role emotional	− 0.502	− 0.435	− 0.307	− 0.459	− 0.377	− 0.316
SF-12: mental health	− **0.611**	− 0.553	− 0.457	− 0.583	− 0.450	− 0.499
SF-12: physical component score	− **0.631**	− 0.493	− 0.308	− **0.725**	− 0.511	− 0.526
SF-12: mental component score	− 0.483	− 0.465	− 0.346	− 0.425	− 0.356	− 0.315
MC-QoL: symptoms	**0.832**	**0.676**	0.486	**0.833**	**0.620**	**0.601**
MC-QoL: social life/functioning	**0.773**	**0.625**	0.506	**0.768**	0.547	**0.604**
MC-QoL: emotions	**0.712**	0.580	0.512	**0.710**	0.493	**0.727**
MC-QoL: skin	**0.635**	0.459	**0.779**	**0.661**	0.397	**0.795**
MC-QoL: total score	**0.849**	**0.679**	0.587	**0.853**	**0.602**	**0.730**
PGIS	**0.618**	0.454	0.446	**0.610**	0.373	0.543

Correlation coefficients ≥ 0.6 = bold; correlation coefficients < 0.3 = italic

ISM-SAF^©^ daily scores used for analyses at Day 15 to match PGIS recall period

ISM-SAF^©^ weekly scores (Days 9–15) used for analyses at Day 15 to match SF-12v2^®^ recall period

ISM-SAF^©^ biweekly mean scores (Days 2–15) used for analyses at Day 15 to match MC-QoL recall period

#### Known-groups analysis

Based on results from both cohorts, TSS, GSS, and SSS scores were clearly distinct across all patient severity groups, in the hypothesized direction (i.e., patients with greater symptoms and impacts, as assessed by the PGIS, MC-QoL, and SF-12v2^®^, also scored higher on the ISM-SAF), and those differences were statistically significant (*p* < 0.05) (Table 5). Additional file 1: Table S4 presents comparable data for those patients with only a self-reported diagnosis.

**Table 5:** Known-groups analysis of the ISM-SAF total and domain scores based on PGIS, MC-QoL, and SF-12v2^®^ assessments administered at Day 15

PRO	Group	Self-reported diagnosis cohort (N = 103)				Medically documented diagnosis cohort (n = 58)			
		n	TSS M (SD)	GSS M (SD)	SSS M (SD)	n	TSS M (SD)	GSS M (SD)	SSS M (SD)
PGIS^a^	Absent/minimal	41	16.5 (14.8)	3.0 (4.8)	5.3 (4.2)	26	18.5 (14.1)	3.9 (5.1)	4.7 (3.9)
	Moderate	43	29.3 (12.5)	5.6 (3.9)	9.2 (5.4)	22	32.4 (13.0)	5.8 (3.7)	10.3 (5.3)
	Severe/very severe	18	48.3 (19.6)	9.6 (7.4)	12.2 (7.0)	9	50.4 (20.7)	9.3 (7.7)	11.8 (5.7)
MC-QoL^b^	Mild	37	13.4 (8.2)	2.3 (2.4)	5.2 (4.3)	23	16.6 (9.9)	3.4 (3.0)	5.4 (4.7)
	Moderate	32	27.9 (8.7)	5.0 (3.1)	9.1 (5.0)	15	29.5 (9.3)	5.1 (3.3)	9.5 (5.4)
	Severe	33	42.2 (13.9)	9.2 (4.6)	10.9 (4.7)	19	42.0 (12.0)	8.3 (3.6)	11.0 (3.3)
SF-12v2^®^^c^	Mild	34	17.8 (15.6)	3.1 (3.5)	7.0 (6.2)	19	17.2 (11.5)	3.1 (2.6)	5.6 (4.4)
	Moderate	33	23.9 (10.2)	4.7 (3.3)	7.2 (4.3)	18	24.8 (8.7)	4.7 (3.5)	8.1 (5.3)
	Severe	34	40.2 (15.0)	8.3 (5.5)	10.8 (4.7)	19	43.9 (13.4)	8.8 (4.2)	11.7 (3.9)

*P* values in Table 5 were < 0.05 for all analyses based upon a Kruskal–Wallis one-way analysis of variance comparing overall difference between median scores of the groups

^a^ISM-SAF^©^ daily scores used for analyses at Day 15 to match PGIS recall period

^b^MC-QoL groups were formed by splitting the sample into tertile groupings (dividing points: 38.9, 55.6) with higher scores indicating greater disease-related impairment. ISM-SAF^©^ biweekly mean scores (Days 2–15) used for analyses at Day 15 to match MC-QoL recall period

^c^SF-12v2^®^ groups were formed by splitting the sample into tertile groupings (dividing points: 31.9, 44.6) with higher scores indicating greater disease-related impairment. ISM-SAF^©^ weekly scores (Days 9–15) used for analyses at Day 15 to match SF-12v2^®^ recall period

### ISM-SAF score severity cutoffs

#### ISM-SAF tertile groupings

The biweekly TSS marking the 33^rd^ percentile (P33) was 19.1 for the Self-reported Diagnosis Cohort and 20.6 for the Medically Documented Diagnosis Cohort. The biweekly TSS scores marking P66 were 31.2 and 35.1, respectively. These results suggest that a biweekly TSS ranging from 19.1 to 20.6 would delineate the two-thirds of the study population reporting the most severe symptomatic experience.

#### ROC curve analysis

**Fig. 1:**
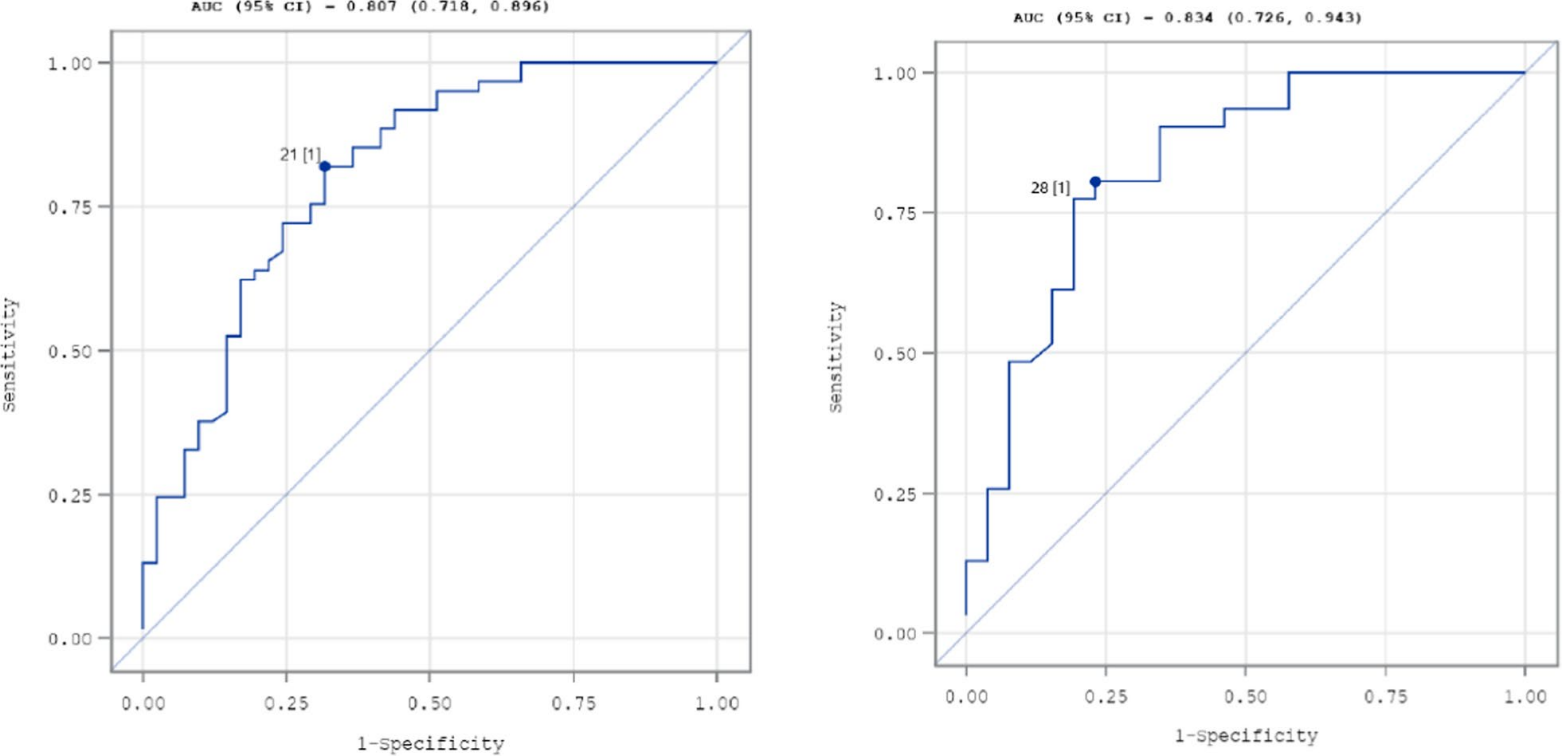
Receiver operating characteristic curve: Biweekly total symptom score predicting moderate/severe/very severe on Patient Global Impression of Severity—Self-reported Diagnosis Cohort (n = 102; **a** shown on the left) and Medically Documented Diagnosis Cohort (n = 57; **b** shown on the right)

The analysis of the Self-reported Diagnosis Cohort suggested a TSS of 21 (sensitivity = 82.0% [i.e., correctly identifies 82.0% of patients with moderate to severe symptoms], specificity = 68.3% [i.e., correctly identifies 68.3% of patients whose symptoms are not moderate or severe], PPV = 79.4% [i.e., correctly identifies 79.4% of patients classified as moderate or severe/very severe on the PGIS], NPV = 71.8% [i.e., correctly identifies 71.8% of patients classified as absent/minimal on the PGIS], Youden's index = 0.50) can be used as a threshold to identify patients with moderate symptoms (Fig. 1a).

The analysis of the Medically Documented Diagnosis Cohort suggested a TSS of 28 (sensitivity = 80.7%, specificity = 76.9%, PPV = 80.6%, NPV = 76.9%, Youden's index = 0.58) can be used as a threshold to identify patients with at least a moderate condition (Fig. 1b).

## Discussion

With its content validity established [14], results from the present observational study demonstrated the ISM-SAF to be capable of generating reliable and construct valid scores when administered in its target patient population. Specifically, internal consistency estimates (α) for the TSS express strong reliability and, while lower for the GSS and SSS, are still acceptable, particularly for a newly developed assessment [21]. Further, test–retest reliability (all ICCs ≥ 0.86), construct validity (e.g., correlational analyses indicated that ISM-SAF scores were more strongly correlated with variables assessing symptoms and physical function, and less strongly correlated with variables associated with more distal disease impacts), and known-groups analyses (e.g., TSS, GSS, and SSS were distinguished among clinically unique groups as specified by the PGIS, SF12v2^®^, and MC-QoL) all generated results supporting the strong performance of the ISM-SAF scores.

Another goal of the study was to estimate a cutoff value for the ISM-SAF TSS capable of distinguishing respondents who experience moderate to severe signs and symptoms of ISM from those who are less afflicted. The purpose of this exploration was to anticipate use of the ISM-SAF to screen patients into (or out of) future clinical studies based on a minimum level of symptom severity. While descriptive tertile groupings suggest a biweekly TSS in the range of 19.1 to 20.6 would delineate patients reporting the most severe experience of ISM symptoms, ROC analyses suggested that a biweekly TSS of 21 to 28 would be adequate for that purpose. Choosing an optimal cutoff point for clinical trial screening purposes, however, should take other factors into consideration. For example, particularly for a rare condition (such as systemic mastocytosis), care must be taken to ensure that the severity cutoff point does not exclude large numbers of potential patients (i.e., does not limit the clinical study sample and ability to draw reliable conclusions regarding product efficacy or safety). For the present study, a biweekly ISM-SAF TSS cutoff value of between 21 and 28 was suggested for screening purposes in Blueprint Medicine’s BLU-285–2203 pivotal Phase 2 clinical trial. The upper value of 28 was the more conservative recommendation, and it was assumed that the use of this cutoff would retain a large enough sample to meet clinical study goals. Researchers could be confident that the use of this cutoff value would allow the identification of patients with moderate to severe symptoms.

Patients who entered this study were taking many concomitant medications. Thus, it should be noted that patients’ symptom experience—as captured by the ISM-SAF—could have been impacted by management of ISM symptoms through the use of symptomatic treatments. Although there is the potential for experience of side effects with medication use, it is anticipated that the overall ISM symptom experience of patients in this study may have been less severe than in the absence of symptomatic treatment use. This further supports the value of 28 as a more conservative recommendation for moderate symptom threshold; however, the relatively small proportion of patients in the more severe PGIS categories should be noted as a limitation to the score severity cutoff analyses.

Although study patients reported symptom severity across the range of ISM-SAF response options (0–10), responses clustered near the lower end of the scale (i.e., less severe symptoms). From a measurement perspective, it is tempting to conclude a “floor effect” (a potentially artificial or unnatural lower limit of response choices and subsequent inability to measure levels of the target concept that fall below that lower limit) [26]. Relevant here, however, is that it is conceptually impossible to experience a symptom less severely than not experiencing the symptom at all (which is what the response choice of “0” reflects, “No [symptom]”). Therefore, it is likely that the observed data reflects the actual experiences of the target patient population, and this was anticipated and consistent with the qualitative research activities that contributed to the development of the ISM-SAF and showed that not all patients experience all symptoms on all days and when they do experience a given symptom, its severity is variable [14].

Another potential limitation in this study is that patients self-reported their ISM or SSM diagnosis. To address the possibility of including patients who did not have systemic mastocytosis, a separate psychometric analysis was performed on the 56% (n = 58/103) of patients who provided medical documentation of a confirmed ISM or SSM diagnosis. The reliability and validity findings were similar between the two cohorts, which adds to investigator confidence that the entire sample (N = 103) did have ISM or SSM. Additionally, psychometric analyses were performed on patients with only a self-reported diagnosis without medical documentation (44%, n = 45/103) to give confidence that the scores were similar between patient samples. While minor differences in the data were observed (e.g., less distinct differences in the SSS between known groups of patients with only a self-reported diagnosis), overall findings from the post-hoc analysis were comparable to those from patients with a medically documented diagnosis. This similarity in demographic characteristics and score reliability and validity estimates supports the conclusion that these two samples come from the same population of patients, demonstrating the veracity of the results.

## Conclusions

In conclusion, the ISM-SAF produced reliable and construct-valid scores that were capable of distinguishing among clinically distinct groups when administered in the target patient population. These results, along with its strong development history including review, comment, and input from division representatives from the FDA and evidence of content validity, support the use of the ISM-SAF in clinical studies designed to evaluate ISM treatments pursuant to product labeling goals. Additionally, this study supported the use of the ISM-SAF as a study entry criteria tool (using a biweekly TSS of between 21 and 28 as a potential cutoff) for future clinical studies. Implementation of the ISM-SAF in future studies will enable further evaluation of the psychometric performance of its scores, including sensitivity to change, as well as inform score interpretation guidelines, when administered to patients with ISM.

## Supplementary Information

**Additional file 1: Table S1.** Sample demographic and health characteristics. **Table S2**. Internal consistency reliability (α) on the biweekly ISM-SAF© total symptom scale and domain scores (Days 2–15). **Table S3.** Spearman correlations of ISM-SAF total and domain scores with other measures administered at Day 15. **Table S4.** Known-groups analysis of the ISM-SAF total and domain scores based on PGIS, MC-QoL, and SF-12v2^®^ assessments administered at Day 15.

## Data Availability

The datasets used and/or analyzed during the current study are available from the corresponding author on reasonable request.

## Abbreviations

FDA: Food and Drug Administration
GSS: Gastrointestinal symptom score
ICC: Intraclass correlation coefficient
IRB: Independent review board
ISM: Indolent systemic mastocytosis
ISM-SAF: Indolent Systemic Mastocytosis Symptom Assessment Form
MC-QoL: Mastocytosis Quality of Life Questionnaire
NPV: Negative predictive value
NRS: Numeric rating scale
PGIS: Patient Global Impression of Severity
PPV: Positive predictive value
PRO: Patient-reported outcome
ROC: Receiver operating characteristic
SD: Standard deviation
SF-12v2^®^: 12-Item Short Form Survey, version 2
SSM: Smoldering systemic mastocytosis
SSS: Skin symptom score
TSS: Total symptom score
