# Supplementary material for: Psychometric evaluation of the Indolent Systemic Mastocytosis Symptom Assessment Form (ISM-SAF©) and determination of a threshold score for moderate symptoms
Source: Orphanet J Rare Dis. 2023 Mar 25;18:69. doi: 10.1186/s13023-023-02661-1 (PMC10039595; doi:10.1186/s13023-023-02661-1)
Supplement: Supplementary file 1 — Additional file 1: Table S1. Sample demographic and health characteristics. Table S2. Internal consistency reliability (α) on the biweekly ISM-SAF© total symptom scale and domain scores (Days 2–15). Table S3. Spearman correlations of ISM-SAF total and domain scores with other measures administered at Day 15. Table S4. Known-groups analysis of the ISM-SAF total and domain scores based on PGIS, MC-QoL, and SF-12v2® assessments administered at Day 15. [file 13023_2023_2661_MOESM1_ESM.docx]

Supplementary Tables

| Supplementary Table 1. Sample demographic and health characteristics | | |
| --- | --- | --- |
| Demographic or health characteristic | Self-reported Diagnosis Without Medical Documentation Cohort (n=45)  Statistic or n (%) | Medically Documented Diagnosis Cohort (n=58)  Statistic or n (%) |
| Age at Day 1 (in years) | | |
| Mean (SD) | 51.9 (11.7) | 48.9 (13.3) |
| Median | 50.4 | 47.4 |
| Min–Max | 28.9–76.1 | 18.6–72.2 |
| Missing/No response | 3 | 4 |
| Gender | | |
| Female | 32 (71.1%) | 52 (89.7%) |
| Male | 13 (28.9%) | 6 (10.3%) |
| Race | | |
| White | 43 (95.6%) | 58 (100.0%) |
| Other^a^ | 2 (4.4%) | 0 (0.0%) |
| Ethnicity | | |
| Hispanic or Latino | 5 (11.1%) | 1 (1.7%) |
| Not Hispanic or Latino | 40 (88.9%) | 57 (98.3%) |
| Work status | | |
| Working full-time | 24 (53.3%) | 21 (36.2%) |
| On disability^b^ | 8 (17.8%) | 11 (19.0%) |
| Working part-time^c^ | 4 (8.9%) | 14 (24.1%) |
| Retired | 7 (15.6%) | 7 (12.1%) |
| Other^d^ | 1 (2.2%) | 3 (5.2%) |
| Unemployed | 1 (2.2%) | 1 (1.7%) |
| Student | 0 | 1 (1.7%) |
| Highest level of education | | |
| High school diploma (or GED) or less | 1 (2.2%) | 2 (3.4%) |
| Some college or certificate program | 14 (31.1%) | 15 (25.9%) |
| College or university degree (two- or four-year) | 20 (44.4%) | 24 (41.4%) |
| Graduate degree | 10 (22.2%) | 17 (29.3%) |

^a^Other race includes patients who characterized themselves as “Hispanic” and “Puerto Rican.”

^b^On disability includes those that are awaiting a disability hearing/decision.

^c^Part-time is characterized as work that is not consistently done five days a week.

^d^Other work statuses include patients who noted that they are self-employed or work from home but did not indicate how much time per week they are working.

| Supplementary Table 2. Internal consistency reliability (α) on the biweekly ISM-SAF^©^ total symptom scale and domain scores (Days 2–15) | | |
| --- | --- | --- |
|  | Self-reported Diagnosis Without Medical Documentation Cohort (n=45) | Medically Documented Diagnosis Cohort (n=58) |
|  | Coefficient alpha^a^ | Coefficient alpha^a^ |
| Domain/Total score^b^ | | |
| TSS | 0.892 | 0.876 |
| GSS | 0.850 | 0.685 |
| SSS | 0.633 | 0.700 |
| Items | Alpha of TSS if item removed | |
| Item 1: Bone pain | 0.878 | 0.862 |
| Item 2: Abdominal pain | 0.875 | 0.859 |
| Item 3: Nausea | 0.880 | 0.861 |
| Item 4: Spots | 0.911 | 0.881 |
| Item 5: Itching | 0.885 | 0.866 |
| Item 6: Flushing | 0.882 | 0.859 |
| Item 7: Fatigue | 0.874 | 0.849 |
| Item 8: Dizziness | 0.877 | 0.859 |
| Item 9: Brain Fog | 0.885 | 0.867 |
| Item 10: Headache | 0.882 | 0.861 |
| Item 11: Diarrhea (frequency) | — | — |
| Item 12: Diarrhea severity | 0.878 | 0.887 |

^a^Only coefficient alpha for the ISM-SAF^©^ domain scores presented (i.e., item to domain score correlation and coefficient alpha with item removed are not presented here). The Cronbach’s alpha presented for each item is the α of the TSS if the item was removed

^b^The ISM-SAF^©^ item score ranges from 0 to 10, while the domain and total scores (GSS, SSS, and TSS) range from 0 to 30, 0 to 30, and 0 to 110, respectively; for all score types, higher scores are associated with a higher level of symptom severity.

| Supplementary Table 3. Spearman correlations of ISM-SAF total and domain scores with other measures administered at Day 15 | | | | | | |
| --- | --- | --- | --- | --- | --- | --- |
| Concurrent measure | Self-reported Diagnosis Without Medical Documentation Cohort (n=45) | | | Medically Documented Diagnosis Cohort (n=58) | | |
|  | TSS | GSS | SSS | TSS | GSS | SSS |
| SF-12: Physical Functioning | -0.457 | -0.418 | *-0.013* | **-0.685** | -0.530 | -0.484 |
| SF-12: Role Physical | **-0.761** | **-0.697** | *-0.250* | **-0.729** | -0.547 | -0.528 |
| SF-12: Bodily Pain | **-0.690** | -0.597 | *-0.248* | **-0.760** | -0.514 | -0.585 |
| SF-12: General Health | -0.452 | -0.404 | *-0.139* | **-0.667** | -0.432 | -0.511 |
| SF-12: Vitality | -0.565 | **-0.602** | *-0.186* | -0.453 | -0.305 | *-0.222* |
| SF-12: Social Functioning | **-0.618** | **-0.669** | *-0.231* | -0.577 | -0.505 | -0.408 |
| SF-12: Role Emotional | -0.557 | -0.535 | -0.303 | -0.459 | -0.377 | -0.316 |
| SF-12: Mental Health | **-0.632** | **-0.652** | -0.417 | -0.583 | -0.450 | -0.499 |
| SF-12: Physical Component Score | -0.510 | -0.449 | *-0.042* | **-0.725** | -0.511 | -0.526 |
| SF-12: Mental Component Score | -0.567 | **-0.613** | -0.382 | -0.425 | -0.356 | -0.315 |
| MC-QoL: Symptoms | **0.829** | **0.754** | 0.363 | **0.833** | **0.620** | **0.601** |
| MC-QoL: Social Life/Functioning | **0.797** | **0.736** | 0.418 | **0.768** | 0.547 | **0.604** |
| MC-QoL: Emotions | **0.727** | **0.673** | 0.352 | **0.710** | 0.493 | **0.727** |
| MC-QoL: Skin | **0.610** | 0.529 | **0.758** | **0.661** | 0.397 | **0.795** |
| MC-QoL: Total Score | **0.844** | **0.773** | 0.438 | **0.853** | **0.602** | **0.730** |
| PGIS | **0.671** | 0.572 | 0.354 | **0.610** | 0.373 | 0.543 |

Note: Correlation coefficients ≥0.6=**green and bold**; correlation coefficients <0.6 and ≥0.3=gray; and correlation coefficients <0.3=*red and italic*

ISM-SAF^©^ daily scores used for analyses at Day 15 to match PGIS recall period

ISM-SAF^©^ weekly scores (Days 9–15) used for analyses at Day 15 to match SF-12v2® recall period

ISM-SAF^©^ biweekly mean scores (Days 2–15) used for analyses at Day 15 to match MC-QoL recall period

| Supplementary Table 4. Known-groups analysis of the ISM-SAF total and domain scores based on PGIS, MC-QoL, and SF-12v2® assessments administered at Day 15 | | | | | | | | | |
| --- | --- | --- | --- | --- | --- | --- | --- | --- | --- |
| PRO | Group | Self-reported Diagnosis Without Medical Documentation Cohort (n=45) | | | | Medically Documented Diagnosis Cohort (n=58) | | | |
|  |  | n | TSS M (SD) | GSS M (SD) | SSS M (SD) | n | TSS M (SD) | GSS M (SD) | SSS M (SD) |
| PGIS^a^ | Absent/Minimal | 15 | 13.1 (15.8) | 1.5 (3.9) | 6.2 (4.7) | 26 | 18.5 (14.1) | 3.9 (5.1) | 4.7 (3.9) |
|  | Moderate | 21 | 26.1 (11.3) | 5.5 (4.3) | 8.0 (5.3) | 22 | 32.4 (13.0) | 5.8 (3.7) | 10.3 (5.3) |
|  | Severe/Very Severe | 9 | 46.1 (19.4) | 9.8 (7.5) | 12.7 (8.5) | 9 | 50.4 (20.7) | 9.3 (7.7) | 11.8 (5.7) |
| MC‑QoL^b^ | Mild | 17 | 11.6 (7.8) | 1.7 (2.5) | 5.4 (4.9) | 23 | 16.6 (9.9) | 3.4 (3.0) | 5.4 (4.7) |
|  | Moderate | 14 | 26.4 (7.1) | 4.3 (2.7) | 8.9 (3.7) | 15 | 29.5 (9.3) | 5.1 (3.3) | 9.5 (5.4) |
|  | Severe | 14 | 42.5 (16.6) | 10.4 (5.6) | 10.8 (6.4) | 19 | 42.0 (12.0) | 8.3 (3.6) | 11.0 (3.3) |
| SF‑12v2®^c^ | Mild | 15 | 18.5 (20.2) | 2.9 (4.3) | 8.8 (7.6) | 19 | 17.2 (11.5) | 3.1 (2.6) | 5.6 (4.4) |
|  | Moderate | 15 | 21.6 (11.6) | 4.1 (3.5) | 6.7 (4.3) | 18 | 24.8 (8.7) | 4.7 (3.5) | 8.1 (5.3) |
|  | Severe | 15 | 37.0 (14.8) | 8.6 (6.3) | 9.1 (4.6) | 19 | 43.9 (13.4) | 8.8 (4.2) | 11.7 (3.9) |

Note: P-values in Supplementary Table 4 were <0.05 for all analyses based upon a Kruskal-Wallis one-way analysis of variance comparing overall difference between median scores of the groups, with the exception of the SSS data for the cohort of patients without confirmed medical documentation (PGIS p=0.07; MC-QoL p<0.05; SF-12v2® p=0.49).

^a^ISM-SAF^©^ daily scores used for analyses at Day 15 to match PGIS recall period

^b^MC‑QoL groups were formed by splitting the sample into tertile groupings with higher scores indicating greater disease-related impairment. ISM-SAF^©^ biweekly mean scores (Days 2–15) used for analyses at Day 15 to match MC-QoL recall period.

^c^SF‑12v2® groups were formed by splitting the sample into tertile groupings with higher scores indicating greater disease-related impairment. ISM-SAF^©^ weekly scores (Days 9–15) used for analyses at Day 15 to match SF-12v2® recall period.
